# The Relationships between High School Subjects in terms of School Satisfaction and Academic Performance in Mexican Adolescents

**DOI:** 10.3390/ijerph16183494

**Published:** 2019-09-19

**Authors:** Raúl Baños, Antonio Baena-Extremera, Antonio Granero-Gallegos

**Affiliations:** 1 Department of Physical Education and Sports Science, Autonomous University of Baja California, 22890 Ensenada, Mexico; raulfb89@gmail.com; 2 Department of Didactic of Corporal Expression, Faculty of Education Sciences, 18071 Granada, Spain; 3 Health Research Center, Department of Education, Faculty of Education Sciences, 04120 Almería, Spain; agranero@ual.es

**Keywords:** academic performance, satisfaction, subjects, secondary education, students

## Abstract

Adolescents’ academic performance and the way it is related to their subjective wellbeing are issues of great interest across educational systems. The purpose of this study was to ascertain how satisfaction with high school subjects can predict school satisfaction and academic performance in Mexican students. The sample consisted of 457 high school students in the Baja California and Nuevo León states in Mexico (247 boys, 210 girls); their mean age being 14.10 (SD = 0.84). We used a questionnaire featuring a subject satisfaction scale, an intrinsic school satisfaction scale, and one related to academic grades. We used descriptive analyses, correlations, and structural regression models. In terms of results, the high satisfaction and academic performance levels in physical education, Spanish and English are worth highlighting. Geography and history are the most relevant predictors of academic grades, while Spanish predicts school satisfaction and physical education predicts boredom. In conclusion, satisfaction with mathematics, Spanish, and English are strong predictors of satisfaction (SATF), and the latter in turn predicts Mexican high school students’ academic performance.

## 1. Introduction

In recent years, studies related to subjective well-being have grown, assessing their relationship with school dropping-out [1], scholar satisfaction [2], and the practice of sporting-physical activity in the leisure time [3], among others. In line with this, the increasing physical inactivity in adolescents is of great concern, being one of the ten main risk factors of death worldwide and responsible for diseases that cause morbidity and mortality according to the World Health Organization [4]. The sedentary lifestyle has been related to different pathologies that harm the quality of life of the human being, associated with diseases such as depression [5], diabetes [6], cardiovascular diseases [7], obesity [8], bipolar disorder [9], and with satisfaction with life [10]. In regard to Mexico, the number of teenagers who report having abandoned physical-sports practice in their free time is bothersome [11]. Relationships between abandoning sports practice and satisfaction with physical education [12], and with academic performance [13] have been found.

The recent low PISA Worldwide Ranking [14] academic performance results obtained by Mexican students establish a serious education problem in the country. Mexico’s academic performance level is well below the Organization for Economic Co-operation and Development countries’ average (OECD), [15]. It obtained an average score of 416 in Science, 423 in Reading, and 408 in Math; the OECD average scores being 493, 493, and 490 respectively for each area [15]. Concerns increase when we observe that the results obtained have shown no significant improvement since 2003 [15]. Thus, it is important to ascertain those aspects which might have an impact on the problem that Mexican adolescents’ academic performance poses.

Subjective wellbeing is one of the variables included in the latest PISA Worldwide Ranking [14] as an important factor in students’ academic performance. Mexican adolescents obtained school-specific wellbeing levels which are well below the OECD countries average [15], a factor likely to affect academic performance. Diener’s subjective well-being theory [16] can be of great help in studying subjective well-being. This theoretical construct is formed by two dimensions, one cognitive (satisfaction in general with life) and another of the affective type (affective balance). The cognitive dimension increases the ability to critically assess your satisfaction with certain vital areas. In line with this, the affective dimension helps by beginning a stage of identity and autonomy search, in which, due to the controversy between their self-management needs and the external barriers they encounter, may trigger an increase in feelings of misunderstanding and dissatisfaction in the family environment and with the academic [17]. Accordingly, satisfaction with the student’s life is understood as a general summary of his life, and the affective balance as the result of the immediate and continuous reactions that happen to the students [18]. If we focus on satisfaction with the school, it would be understood as a cognitive–affective evaluation of the satisfaction that the student experiences with their own school experience [19] and with the courses [20].

School-specific subjective wellbeing is defined as the subjective and cognitive assessment of quality of life in the school environment [21,22]. These assessments, whether positive or negative, include judgment and feelings around interest, commitment and affective reactions, such as joy and sadness in relation to school events [23]. School satisfaction is key to adolescents’ development as it is associated with high levels of life satisfaction [24], social relationships [25], academic commitment [26], adequate school climate [27], and academic success [28], among other variables. By contrast, boredom at school hinders the efficiency of all learning strategies [29], which affects adolescents’ academic performance [30], and is related to high levels of early school leaving and depression [31], and high stress and anxiety levels [32]. As an example of the last effect, the OECD found that Mexican adolescents had higher levels of school anxiety than the OECD average [15]. This is worrying data which are likely to have significant impacts, both in terms of students’ academic performances and their health.

As different studies reveal, students’ levels of satisfaction in the school context can be analyzed from different areas. Baños, Ortiz-Camacho, Baena-Extremera, and Tristán-Rodríguez [33] claim that the satisfaction experienced by students with each high school subject can have an impact on school-specific subjective wellbeing. In line with this, Baños, Baena-Extremera, and Ortiz-Camacho [20] found that both satisfaction and boredom in physical education (PE), geography and history (GH), English (ENG), Spanish (SP), and mathematics (MATH) predicted school satisfaction and boredom, in the context of the Spanish educational system. However, they did not find strong predictions based on academic performance, and no predictions based on boredom were observed [20].

If we focus on students’ satisfaction with PE, physical education does predict school satisfaction [34], and adolescents who have more weekly PE hours have higher cognitive performance [35]. In line with this, scientific evidence has proved that boredom in PE is a strong predictor of school boredom [20,34] and is related to students’ abandoning the leisure physical activity [36]. Increasing that concern that this evokes is the fact that physical inactivity has been related to depressive behaviors [37].

Regarding GH, the literature reveals that satisfaction with this subject positively predicts school satisfaction and negatively predicts boredom in Spanish students [20]. Furthermore, the importance of a GH and MATH learning process which is in keeping with the pace of development of the intellectual skills of each student, to avoid dissatisfaction with these subjects, is worth noting here [38].

The high level of satisfaction and academic performance in the case of ENG is related, according to Joo and Lim [39], to the financial efforts that governments have made to open new bilingual secondary schools. Together with an increase in the number of bilingual schools, there has been more innovation in electronic learning resources, which promotes autonomous learning and increases students’ motivation [39,40]. Furthermore, some authors claim that economic investment in facilities and teaching materials in bilingual schools is associated with an increase in learning satisfaction and predicts achievements in ENG, SP, and MATH [41].

With regard to boredom in SP, the literature shows that it is related to perceptions regarding teachers’ skills, given that teachers do not promote creativity through motivational strategies [38], thus slowing the pace of learning [42]. However, when students find SP interesting, they show more commitment to tasks and fewer detachment behaviors [43]. Moreover, when adolescents have a high self-confidence with their learning of a subject and attribute their results to internal and manageable causes, their academic performance improves, as seen in Miñano and Castejón [44].

Finally, with regard to MATH, students’ satisfaction in this subject is related to the high ease of learning and low anxiety levels. On the other hand, boredom in MATH relates to high dropout rates [45], and learning difficulties, which causes anxiety in MATH learning in adolescents [46], with anxiety increasing even more during exams [47]. It is important that students do not experience boredom in school, as it is related to lower levels of mental health and the general well-being of students [48]. About that, it is necessary to specify that boredom is identified with student dissatisfaction [16], and it depends on themselves and their learning experience; it is not only because of the teachers’ performances.

The research reveals the importance of students being satisfied with the different subjects in the secondary school syllabus. For example, the PISA Worldwide Ranking [14] results obtained by Spanish adolescents have been improved from the start so that this Spanish speaking country now ranks above the OECD countries’ average. By contrast, Mexican adolescents’ results have seen no improvement since 2003. Mexican students’ poor academic performance and school dissatisfaction [15] has led us to question ourselves in relation to students’ feelings of satisfaction with school subjects. Thus, the hypothesis in this study is that all subjects predict school satisfaction and that the latter, in turn, predicts academic performance to a greater or lesser extent. Taking this into account, the object of this study was to ascertain how satisfaction with high school subjects can predict school satisfaction and academic performance in Mexican students.

## 2. Materials and Methods

### 2.1. Design and Type of Research

The design featured in this study is non-experimental, quantitative, and cross-sectional as the variables were not manipulated, and only the psychometric properties of the instruments analyzed were identified. Furthermore, it is a predictive cross-sectional study, given that its object is to analyze functional relationships through the prediction of a variable based on one or more predictors [49]. This study was carried out in accordance with the Declaration of Helsinki in 1961 (revised in Edinburgh in 2000). Approval was obtained by the Secretaria de Educación Pública of Mexico and Universidad Autónoma of Baja California, Mexico (identification number: 431/569/E). 

### 2.2. Participants

We used a non-probabilistic convenience sample, which included 457 high school students from the Nuevo León, San Luis Potosí, and Baja California states (Mexico). The mean age (*M*) was 14.10, with a 0.84 SD. 54% (*n* = 247) of the sample were girls (*M* = 14.01; *SD* = 0.73), and 46% (*n* = 210) were boys (*M* = 14.21; *SD* = 0.94). Furthermore, 47.9% (*n* = 219) were second year high school students, 30.2% (*n* = 138) were third year students, and 21.9% (*n* = 100) were first year students. 

### 2.3. Instruments

The intrinsic satisfaction scale adapted to the subjects of secondary school: EF, GH, ENG, ESP, and MAT. The Intrinsic Satisfaction Classroom, ISC was adapted to high school PE, GH, ENG, SP, and MATH by Baños et al. [20] based on the original by Baena-Extremera, Granero-Gallegos, Bracho-Amador, and Pérez-Quero [50]. It was used to assess satisfaction with school subjects. This instrument was adapted to the Mexican context, as it had been validated for the Spanish one. The instrument is composed of eight items; five of them measure satisfaction/fun level and three items measure boredom with the academic activities of each subject. The scale was preceded by the sentence, “According to the subject I teach in class, state your level of agreement or disagreement with the following statements.” The answers were collected on a polytomous-item scale from 1 (*totally disagree*) to 5 (*totally agree*). Baños et al. [20] obtained the following internal consistency values: Satisfaction/fun in PE, α = 0.89; boredom in PE, α = 0.79; satisfaction/fun in GH, α = 0.84; boredom in GH, α = 0.75; satisfaction/fun in ENG, α = 0.88; boredom in ENG, α = 0.77; satisfaction/fun in SP, α = 0.86; boredom in SP, α = 0.78; and satisfaction/fun in MATH, α = 0.87; boredom in MATH, α = 0.77.

The Spanish version of the Intrinsic Satisfaction Classroom Scale (*Satisfacción Intrínseca con la Escuela*), adapted to Spanish by Castillo, Balaguer, and Duda [51], based on the original by Duda and Nicholls [52], was used to assess school satisfaction and boredom. The scale was adapted to the Mexican context given that it had been validated for the Spanish one. The instrument is composed of eight items that measure satisfaction (SATF) levels (five items) and boredom (BORD) levels (three items). The scale was preceded by the sentence, “Please state your level of agreement or disagreement with the following statements related to all your school subjects.” The answers were collected on a polytomous-item scale from 1 (totally disagree) to 5 (totally agree). In Baena-Extremera, Granero-Gallegos and Ortiz-Camacho [53], internal consistency values were satisfaction/fun: α = 0.73; boredom: α = 0.76.

Finally, we requested from teachers their records of the latest test scores in order to analyze students’ grades. This procedure is more objective than the one in Baños et al. [20], as these authors asked the students themselves for their grades. They were recorded on a polytomous-item scale ranging from 1 to 10. 

### 2.4. Procedure

We requested permission from the high schools’ management prior to carrying out the research and provided students’ parents/guardians with an informed consent form stating the objectives of the research. Once permission was obtained, we collected the data and informed students on the object of the study, its anonymous and voluntary nature, and the confidentiality of their answers. They were also told that there were no correct or incorrect answers and they were requested to answer with maximum sincerity. The questionnaires were filled out in the classroom with the main researcher available at all times to resolve doubts about the process, which took 30–40 min.

### 2.5. Statistical Analysis

First, we carried out a descriptive analysis of all the items in the scales and sub-scales.

More on that, analyses of the normality of data were carried out using the PRELIS relative multivariate kurtosis (RMK) test. Once we observed that the data had failed the normality test, we used the weighted least squares (WLS) method in LISREL 8.80 [54]. Then the internal consistency of each variable was calculated with Cronbach’s alpha, composite reliability, average variance extracted (AVE), and McDonald’s omega (Ѡ). Following that, we carried out a confirmatory factor analysis (CFA) for the satisfaction scale adapted to each of the subjects (PE, GH, ENG, SP, and MATH) in order to verify the fit indices in the Mexican context. Following Bentler [55], and Markland [56], the following absolute values are taken into account: *p*-value, associated with the Chi-squared statistic (χ^2^); the χ^2^ and degree of freedom ratio (df; χ^2^/df); GFI (goodness of fit index) and IFI (fit index). As to relative indices, NFI (normed fit index), NNFI (non-normed fit index) CFI (comparative fit index) and root mean square residual (RMSR) were taken into account. Further to that, RMSEA (root mean square error of approximation) was taken into account as an incremental fit index.

Following that, we used structural equation modeling (SEM) in order to test the hypothesized model. The model’s goodness of fit was analyzed through different fit indices: Chi-squared value divided by degrees of freedom (χ^2^/df), non-normed fit index (NNFI), comparative fit index (CFI), and root mean square error of approximation (RMSEA). The estimated parameters were considered significant when the value associated with the *t* value was higher 1.96 (*p* < 0.05). The calculations were carried out with SPSS Statistics V22.0 and LISREL 8.80. 

## 3. Results

### 3.1. Confirmatory Factor Analysis

The psychometric properties of the Satisfaction questionnaire’s dimensions in each of the subjects were studied based on the initial theoretical proposal in Duda and Nicholls [52] and Baños et al. [20]. The confirmatory study was carried out through an analysis of the multivariate normality of each scale. The PRELIS relative multivariate kurtosis (RMK) test in LISREL 8.80 was also carried out. Table 1 shows the values of each subject’s adaptation of the satisfaction questionnaire in the Mexican context. The results reveal that the data is non-parametric. 

Once the results of the normality analysis were obtained, we used WLS of the LISREL 8.80 [54]. The asymptotic variance correlations matrices were also calculated for each of the subjects and were used as input for data analysis. Table 2 shows the CFA results of the different models; all of them are good fit indices, with fit values in terms of χ^2^/df [57,58], GFI [59], CFI, IFI, NFI, NNFI, RMSR [60], and RMSEA [61,62]. Overall, the MATH and GH models show the best fit indexes.

### 3.2. Reliability and Validity Analysis

Table 3 below shows the mean and SD of each subject grade, plus a reliability and validity analysis of each model with Cronbach’s alpha (α) values, composite reliability, the average variance extracted (AVE), and McDonald’s Omega (Ѡ). All the reliability indices, AVE, and virtually all α values are above the acceptable values from Dunn, Baguley, and Brunsden [63] and Hair, Black, Babin, and Anderson [64]. Although some α dimensions obtained values below 0.70, the fact that they have a small number of items per factor (as is the case of the boredom dimensions), means that these α values are acceptable [65]. Furthermore, Vandenbosch [66] claims that composite reliability is considered more adequate than Cronbach’s alpha in ordinal scales given that it does not depend on the number of attributes associated with each concept. We have also obtained acceptable results with McDonald’s Omega [67], which should be between 0.70 and 0.90 [68], though under certain circumstances, values higher than 0.65 can be accepted [69].

The grades’ mean analysis reveals that PE is the subject with the highest mean (*M* = 8.82), and MATH the lowest (*M* = 7.33). As to both of the factors—satisfaction in all subjects and school satisfaction—the values obtained in school satisfaction and boredom are worth highlighting: SATF (*M* = 3.43; *SD* = 0.76) and BORD (*M* = 3.12; *SD* = 0.92). Both are high values, like the academic grade (*M* = 7.93; *SD* = 0.97). In terms of the satisfaction with subjects variables, PE obtained the highest mean values (*M* = 3.94; *SD* = 0.96), followed by GH (*M* = 3.28; *SD* = 0.98), SP (*M* = 3.08; *SD* = 0.98), MATH (*M* = 2.73; *SD* = 0.97), and ENG (*M* = 2.67; *SD* = 1.00). The subject that obtained the highest mean values in the boredom variable was ENG (*M* = 3.08; *SD* = 1.15), followed by MATH (*M* = 3.01; *SD* = 1.12), GH (*M* = 2.75; *SD* = 1.15), SP (*M* = 2.48; *SD* = 1.08), and PE (*M* = 2.00; *SD* = 1.05).

### 3.3. Correlation Analysis

The correlation analysis reveals that each subject satisfaction has significant and negative correlations with the opposite dimension of its scale, and with the school one. On the other hand, it shows statistically significant and positive correlations with school satisfaction in all subjects, the highest being MATH (r = 0.331 **) and the lowest being PE (r = 0.134 **). It also showed significant statistically positive correlations with academic grades, the highest values being for ENG (r = 0.316 **) and MATH (r = 0.175 **); no significant statistically relationship was found between grades and satisfaction with PE. In the case of boredom, GH shows the highest significant statistically correlations with school boredom (r = 0.325 **), the highest negative correlation between subject boredom and school being in ENG (r = −0.212 **). Finally, boredom in ENG has a significant statistically negative correlation with academic grades (r = −0.218 **) (Table 4).

### 3.4. Predicting School Satisfaction and Academic Grades

A series of models was hypothesized in order to achieve the object of this research, one model for each subject, so as to test which had the best goodness of fit, following Markland [56]. As noted before, we tested the satisfaction scale for each of the subjects in this study, satisfaction and boredom being the exogenous variables for each subject, and satisfaction, boredom, and academic grades being the endogenous variables. The structural regression models were analyzed by combining the fit indices mentioned before: χ^2^/df, GFI, CFI, IFI, NFI, NNFI, RMSEA, ECVI (expected cross validation index), and the Akaike information criterion (which deals both with the goodness of fit and simplicity of the model). Table 5 shows that the results obtained in each structural equation model perfectly fit the parameters and can be considered acceptable [57,58,59].

**Figure 1:**
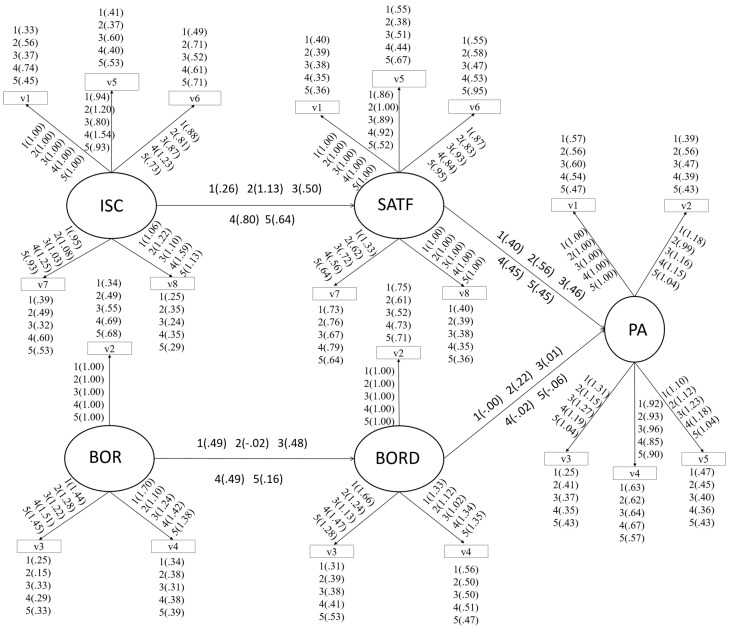
Path diagram for all subjects. Note: Physical education (PE) (1), geography and/or history (GH) (2), English (ENG) (3), Spanish (SP) (4), and mathematics (MAT) (5); ISC: Intrinsic satisfaction classroom with subjects; PE: Boredom with subjects; SATF: Satisfaction with school; BORD: Boredom with school; PA: Performance, academic.

The ECVI in each model reveals that the ENG model is the one obtaining the worst results, followed by the PE and GH models. Furthermore, the Akaike information criterion (AIC) can also be used to compare models, with ENG _(AIC = 608.031)_ and PE _(AIC = 543.230)_ showing the worst results in relation to the other models. However, it is worth noting that the MATH model obtains better χ^2^/df values, while its RMSEA is the same as the rest of the models, except for ENG. The modification index of some models suggested carrying out further predictions for better goodness of fit; e.g., between school satisfaction and grades in MATH (MATH Model); or allowing for some covariance error (models for GH and SP). The path diagram shows gamma, beta, lambda-x, lambda-y, theta delta, and theta epsilon (Figure 1).

Figure 1 show that the models which best predict school satisfaction are SP (γ = 0.80), MATH (γ = 0.64), and ENG (γ = 0.50). On the other hand, the model which best predicts grades based on school satisfaction is GH (β = 0.56), followed by ENG (β = 0.46), and finally PE (β = 0.40). As for the boredom variable, PE and SP (γ = 0.49), and ENG (γ = 0.48) are the subjects with the highest prediction values. It is interesting to note that boredom does not significantly predict academic grades. Finally, it is worth noting that high standardized factor loads were λ > 0.50 and were statistically significant (*t*-value > 1.96) in all the models.

## 4. Discussion

The aim of this study was to assess the way satisfaction with high school subjects can predict school satisfaction and academic performance in Mexican students. It is worth noting here that all the satisfaction with high school subject questionnaires adapted to the Mexican context obtained valid and reliable results, much like the study carried out by Baños et al. [20] in the Spanish context. Regarding intrinsic satisfaction mean values, PE was the subject with the highest scores, followed by GH, SP, MATH, and ENG. MATH, on the other hand, obtained the highest scores in boredom, followed by GH, ENG, SP, and PE.

These results match with those found for Spanish students, who also stated that PE was the subject that gave them most satisfaction and MATH obtained the highest boredom score [20]. These results reveal the importance of PE in the Mexican education system, given that it is the subject that students most enjoy and find the least boring, both being important for the mental health of Mexican adolescents [11].

One reason for concern in relation to Mexican high schools is that boredom levels are higher than enjoyment levels in MATH and ENG. Students’ boredom in these subjects could be related to low investment in updating materials and school facilities [41] and to teachers’ moods [70], as unsatisfied teachers will trigger negative emotions in adolescents [71]. Students’ attitudes as they are, increases the probability that those Mexican adolescents wo; suffer some type of mental illness, since discontent or boredom with school is related to the mental health and general well-being of students [48].

As to the academic performance variable, PE obtained the highest mean values, followed by GH, SP, ENG, and MATH. Spanish adolescents obtained similar results and in the same order of subjects [20]. Academic performance is associated with school satisfaction [33], and with students’ motivational achievements [72]; demotivation can even result in truancy [73,74]. Furthermore, low academic performance is related to fewer possibilities of finding employment in the future [55].

SATF had a positive, significant correlation with MAT, ENG, GH, SP, and PE, ranked by level of significance. However, in Spanish adolescents, SATF had a stronger correlation with PE and was less significant in relation to GH [20]. Other studies also obtained a positive and significant relationship between SATF and PE [34,53]. 

As regards academic performance, it had a positive and significant correlation with satisfaction with ENG, MATH, GH, and SP; there was no relationship with PE, and it had a negative significant relationship only with boredom in ENG. Baños et al. [20] did find a relationship with academic performance, nor with satisfaction with SP. Thus, it would be interesting to continue to study the relationship of satisfaction with high school subjects and academic performance.

The initial hypothesis held that all the subjects would act as school satisfaction predictors and that in turn school satisfaction would predict academic performance. In response to this hypothesis, the path diagram shows that SP is the highest predictor of SATF, followed by MATH, ENG, PE, and GH. These are interesting results, as the three highest predictors of SATF are those which obtained the lower mean satisfaction values, and it is worth noting that ENG and MATH obtained higher levels of boredom than satisfaction. These results could be related to the low school-specific wellbeing levels observed in Mexican adolescents [15], a factor what could explain the low performance in the PISA Worldwide Ranking areas of assessment (reading, science, and math). The data in this research is not encouraging for high school education in Mexico, as if students do not find satisfaction in MATH, learning will become more difficult and anxiety levels will increase [29,46]. Something similar happens with SP; when adolescents do not find this subject interesting, their level of commitment to the tasks and to the subject itself decreases [43]. In addition, boredom in school has been associated with high-risk behaviors, such as drinking, drug use, car racing, and criminal actions [75,76]. It is thus important to prevent students’ from having a low commitment in the classroom, as it increases the probability of early school leaving [77].

GH is the subject which best predicts academic performance based on school satisfaction, followed by ENG, MATH, SP, and PE, though it is worth noting that all subjects obtained similar predictive values. These results reveal the importance for students to feel satisfied with all the subjects for the achievement of good academic performance. However, the low scores obtained by Mexican adolescents in the three areas of the PISA report [14] could be explained by the low satisfaction levels obtained in SP, MATH, and ENG. Aspects such as incompetent SP and MATH teachers and a lack of motivational strategies [19] lead to a slowdown in the pace of learning [42]. This, in turn, leads adolescents to attribute their poor results to external causes, which lowers their academic performance [44]. In this regard, Koroboca and Starobin [28] highlight the importance of students feeling satisfaction with school, so that they are more likely to achieve academic success.

PE and SP, followed by ENG, were the strongest predictors of boredom; no predicting results were obtained with MATH and GH and we did not obtain academic performance prediction values based on boredom. Similar results were obtained with Spanish adolescents [20]. PE was also the best predictor of boredom, and no significant academic performance prediction values based on boredom were observed. PE plays an important role in the educational system, given that adolescents who get bored in the PE class are more likely to feel school boredom too, and consequently, are more likely to drop school or suffer depression [31]. However, the low or null boredom and academic performance predicting value of MATH and GH raises concern. Thus, our initial hypothesis is confirmed by the fact that grades are predicted to varying degrees depending on each subject.

As a limitation of the study, it is worth noting that a non-probabilistic type of sampling has been used so that the results cannot be generalized. It would be interesting to conduct a study where the sample selection is randomized, multi-stage and uses proportional affixation for future research. Another limitation to mention is that the design of the study leads to the potential of inverse causality, since students who have higher academic performance may be more likely to perceive greater satisfaction with subjects and school. Additionally, it did not consider any potential confounding factors, such as socioeconomic status and family environment. More studies should be carried out in which other research designs are proposed, such as experimental studies with intervention programs to reduce boredom with each of the courses.

## 5. Conclusions

In conclusion, satisfaction with MATH, SP, and ENG are strong predictors of SATF, and the latter in turn predicts Mexican high school students’ academic performance. However, the satisfaction levels with these three subjects are the lowest in relation to the rest of the academic subjects. This factor should be taken into account in view of the low PISA Worldwide Ranking [14] results obtained, in terms of academic assessment and school-specific wellbeing, which ranks Mexico as one of the lowest scoring OECD countries. It is worth noting here that PE and GH also predict SATF, which in turns predicts academic performance, though with lower prediction values. We believe it would be interesting in future research to study the level of competence that students perceive in their MATH, SP, and ENG teachers, whether or not teachers use different teaching and motivational strategies, and whether the teaching–learning process matches the intellectual needs of each student, given that those variables have been proven to be related to better satisfaction levels with these subjects and to academic performance [38,42].

## Figures and Tables

**Table 1:** Values of multivariate normality test.

	Multivariant Normalized Kurtosis	Mardia-Based-Kappa	Higher Limit	Lower Limit
ISC-PE	24.134	0.357		
ISC-GH	6.422	0.095		
ISC-ENG	4.529	0.067	1.042	0.958
ISC-SP	3.786	0.056		
ISC-MAT	3.515	0.052		
ISC	9.464	0.140	1.042	0.958

Note: ISC: Intrinsic satisfaction classroom; PE: Physical education; GH: Geography and/or history; ENG: English; SP: Spanish; MAT: Mathematics.

**Table 2:** Models fit index.

	x^2^	gl	x^2^/gl	p	GFI	CFI	IFI	NFI	NNFI	IC90% RMSEA	RMSR	ECVI
ISC-PE	34.30	19	1.80	0.016	0.99	0.97	0.97	0.94	0.96	0.042 [0.02;0.06]	0.04	0.150
ISC-GH	25.89	19	1.36	0.133	0.99	0.98	0.98	0.95	0.98	0.028 [0.00;0.05]	0.05	0.131
ISC-ENG	47.59	19	2.50	0.000	0.98	0.97	0.97	0.94	0.95	0.057 [0.04;0.08]	0.06	0.179
ISC-SP	32.70	19	1.72	0.026	0.99	0.98	0.98	0.95	0.97	0.040 [0.01;0.06]	0.05	0.146
ISC-MAT	20.41	19	1.07	0.370	0.99	0.99	0.99	0.97	0.99	0.013 [0.00;0.06]	0.04	0.119
ISC	20.49	13	1.57	0.083	0.99	0.98	0.98	0.94	0.96	0.036 [0.00;0.04]	0.04	0.111

Note: ISC: Intrinsic satisfaction classroom; PE: Physical education; GH: Geography and/or history; ENG: English; SP: Spanish; MAT: Mathematics; GFI: Goodness of fit index; CFI: Comparative fit index; IFI: Adjustment index; NFI: Normalized adjustment index; NNFI: Non-normative adjustment index; RMSR: Root mean square residual; RMSEA: Root-mean squared approximation error; ECVI: Expected cross validation index.

**Table 3:** Scale of reliability and composite validity.

	M_(Subject grade)_	SD_(Subject grade)_	M	SD	Composite Reliabilty	*AVE*	α	Ѡ
*SATPE*	8.82	1.21	3.94	0.96	0.89	0.63	0.78	0.78
*BORPE*			2.00	1.05	0.77	0.55	0.65	0.76
*SATGH*	8.04	1.30	3.22	0.98	0.83	0.50	0.74	0.81
*BORGH*			2.75	1.15	0.85	0.66	0.70	0.65
*SATENG*	7.47	1.52	2.67	1.00	0.88	0.59	0.78	0.78
*BORENG*			2.74	1.03	0.82	0.60	0.65	0.65
*SATSP*	7.99	1.49	3.08	0.98	0.83	0.50	0.70	0.87
*BORSP*			2.48	1.10	0.78	0.55	0.65	0.71
*SATMAT*	7.33	1.32	2.73	0.97	0.89	0.62	0.75	0.79
*BORMAT*			3.01	1.12	0.81	0.51	0.65	0.71
*SATF*			3.42	0.76	0.76	0.51	0.66	0.79
BORD			3.11	0.92	0.71	0.50	0.65	0.80

Note: M—mean; SD—standard deviation; AVE—average variance extracted; α—Cronbach’s alpha; Ѡ—McDonald’s omega; SATPE—satisfaction with physical education; BORPE—boredom with physical education; SATGH—satisfaction with geography and/or history; BORGH—boredom with Geography and/or history; SATENG—satisfaction with English; BORENG—boredom with English; SATSP—satisfaction with Spanish; BORSP—boredom with Spanish; SATMAT—satisfaction with mathematics; BORMAT—boredom with mathematics; SATF—satisfaction with school; BORD—boredom with school.

**Table 4:** Correlation between satisfaction in subjects, students, and academic grades.

	SATPE	BORPE	SATGH	BORGH	SATENG	BORENG	SATSP	BORSP	SATMAT	BORMAT
SATF	0.134 **	−0.121 **	0.281 **	−0.112 *	0.303 **	−0.212 **	0.242 **	−0.105 *	0.331 **	−196 **
BORS	−0.043	0.226 **	−0.220 **	0.325 **	−0.130 **	0.229 **	−0.224 *	0.278 **	−0.195 **	0.211 **
NOT	0.075	−0.009	0.155 **	−0.073	0.316 **	−0.218 **	0.140 **	−0.0.38	0.175 **	−0.088

Note: SATPE—satisfaction with physical education; BORPE—boredom with physical education; SATGH—satisfaction with geography and/or history; BORGH—boredom with geography and/or history; SATENG: satisfaction with English; BORENG—boredom with English; SATSP—satisfaction with Spanish; BORSP— boredom with Spanish; SATMAT—satisfaction with mathematics; BORMAT—boredom with mathematics. ** *p* < 0.01; * *p* < 0.05.

**Table 5:** Models fit index.

	x^2^	gl	x^2^/gl	p	GFI	CFI	IFI	NFI	NNFI	RMSEA	ECVI	Akaike
ISC PE	449.23	163	2.75	0.000	0.97	0.92	0.92	0.88	0.91	0.06	1.191	543.230
ISC GH	444.77	163	2.73	0.000	0.97	0.92	0.92	0.87	0.90	0.06	1.182	538.767
ISC ENG	514.03	163	3.15	0.000	0.96	0.90	0.90	0.85	0.87	0.07	1.333	608.031
ISC SP	431.09	163	2.64	0.000	0.96	0.90	0.90	0.85	0.88	0.06	1.152	525.090
ISC MAT	425.16	163	2.61	0.000	0.97	0.91	0.91	0.87	0.90	0.06	1.139	519.159

Note: ISC—intrinsic satisfaction classroom; PE—physical education; GH—geography and/or history; ENG:—English; SP: Spanish; MAT—mathematics; GFI—goodness of fit index; CFI—comparative fit index; IFI—adjustment index; NFI—normalized adjustment index; NNFI—non-normative adjustment index; RMSEA—root-mean squared approximation error; ECVI—expected cross validation index.
